# Single-Layer GaInSe_3_: Promising Water-Splitting Photocatalyst with Solar Conversion Efficiency over 30% from Theoretical Calculations

**DOI:** 10.3390/molecules28196858

**Published:** 2023-09-28

**Authors:** Li-Li Liu, Ru-Fei Tang, De-Fen Li, Ming-Xia Tang, Bing-Zhong Mu, Zheng-Quan Hu, Shi-Fa Wang, Yu-Feng Wen, Xiao-Zhi Wu

**Affiliations:** 1 College of Teacher Education, Chongqing Three Gorges University, Chongqing 404100, China; TangRFsanxu@163.com (R.-F.T.); LiDFsanxu@163.com (D.-F.L.); tangmxsanxu@163.com (M.-X.T.); m15213132982@163.com (B.-Z.M.); 2 Institute for Structure and Function, Chongqing University, Chongqing 401331, China; xiaozhiwu@cqu.edu.cn; 3 College of Electronic and Information Engineering, Chongqing Three Gorges University, Chongqing 404100, China; hzq250314@163.com (Z.-Q.H.); wangshifa2006@yeah.net (S.-F.W.); 4 School of Mathematical Sciences and Physics, Jinggangshan University, Ji’an 343009, China

**Keywords:** 2D material, single-layer GaInSe_3_, water-splitting, strain engineering, solar energy

## Abstract

Hydrogen energy from solar water-splitting is known as an ideal method with which to address the energy crisis and global environmental pollution. Herein, the first-principles calculations are carried out to study the photocatalytic water-splitting performance of single-layer GaInSe_3_ under biaxial strains from −2% to +2%. Calculations reveal that single-layer GaInSe_3_ under various biaxial strains has electronic bandgaps ranging from 1.11 to 1.28 eV under biaxial strain from −2% to +2%, as well as a completely separated valence band maximum and conduction band minimum. Meanwhile, the appropriate band edges for water-splitting and visible optical absorption up to ~3 × 10^5^ cm^−1^ are obtained under biaxial strains from −2% to 0%. More impressively, the solar conversion efficiency of single-layer GaInSe_3_ under biaxial strains from −2% to 0% reaches over 30%. The OER of unstrained single-layer GaInSe_3_ can proceed without co-catalysts. These demonstrate that single-layer GaInSe_3_ is a viable material for solar water-splitting.

## 1. Introduction

With the development of human society and economy, the demand and consumption of energy have become unprecedented, and the environmental problems caused by the exploitation and consumption of traditional energy are prominently increasing. Therefore, it is urgent to seek a new source of energy which does not produce pollution in the production and utilization processes. Hydrogen with high calorific value and pollution-free combustion has become the first choice for future energy. Hydrogen energy from efficient and environmentally friendly solar water-splitting has become an ideal method by which to solve our energy-related problems. During the early years of this research, semiconductor oxides such as TiO_2_, ZnO, and SnO_2_ were focused on [1,2,3]. However, the solar-to-hydrogen (STH) conversions of these semiconductor oxides are very limited due to the high recombination rates of charge carriers and low sunlight absorption [4,5]. Hence, reducing the recombination rates of carriers and enhancing solar absorption are two main approaches to improving STH conversions. As compared with traditional semiconductor oxides, two-dimensional (2D) materials possess low carrier recombination rates due to shorter carrier migration distances [6,7,8]. Meanwhile, for 2D materials, since the active potential is rich, the performance of sunlight absorption is strong, and the structural characteristics (such as planar, curved, vertically symmetric, or asymmetrical structures) are diverse, two-dimensional layered materials have been rapidly developed in photocatalytic water-splitting for hydrogen production [6,7,8]. Since solar absorption is directly related to the bandgap, reducing the bandgap is an effective approach to enhancing optical absorption. After the inclusion of electric dipoles (fields), the bandgaps of 2D photocatalysts may decrease to ~0.5 eV [9]. Hence, the inside electric field not only enhances the optical absorption but also effectively reduces the recombination of carriers [10,11].

Single-layer M_2_X_3_ (M = Ga, In; X = S, Se, Te) are a typical group of 2D semiconductors with inside electric fields [12,13]. In_2_X_3_ (X = S, Se, Te) is an indirect bandgap semiconductor with good performance in photocatalytic water-splitting. Single-layer Ga_2_X_3_ (X = S, Se, Te) has better performance of in water-splitting and higher hydrogen production. It has been found that single-layer M_2_X_3_ (M = Ga, In; X = S, Se, Te) has excellent solar water-splitting performance owing to separated charge carriers [10,11]. After the anions in single-layer M_2_X_3_ are replaced, the STH conversion is enhanced [9]. Moreover, the bandgap, band edge position, and sunlight absorption of single-layer chalcogenide can be adjusted via biaxial strain, thereby improving the solar energy conversion efficiency [1,2,3]. In recent years, due to the successful synthesis of vertically asymmetric monolayers Janus MoSSe [14], Janus 2D materials have become a new research hotspot in the field of photocatalysis. The structural, electronic, and transport properties of single-layer GaInX_3_ (X = S, Se, Te) were predicted via first-principles calculations [15]. Single-layer GaInX_3_ were found to have structural stability, highly directional isotropic elasticity, a distinct vacuum level difference, and highly directional isotropic mobility; hence, they are candidates for applications in nanoelectronic devices [15]. In particular, we should note that a direct bandgap of 1.20 eV for single-layer GaInSe_3_ indicates its visible and infrared light absorption [15]. Moreover, as a sulfur-free compound, single-layer GaInSe_3_ is environmentally friendly. Consequently, it would be meaningful to investigate the solar water-splitting performance of single-layer GaInSe_3_.

In this work, the solar water-splitting property of single-layer GaInSe_3_ is calculated by the first-principles method. Since water-splitting can be effectively affected via biaxial strain [16,17], we also apply biaxial strains from −2% to 2% to tune the solar water-splitting property. Theoretical calculations suggest that under biaxial strains from −2% to 0%, single-layer GaInSe_3_ has appropriate band edges for water-splitting and visible optical absorption up to ~3 × 10^5^ cm^−1^. Impressively, the STH efficiency of single-layer GaInSe_3_ under biaxial strains from −2% to 0% surpasses 30%, demonstrating the potential applications of single-layer GaInSe_3_ as a solar water-splitting material.

## 2. Computational Details

First-principles calculations are performed within the framework of the density functional theory (DFT) via the Vienna ab initio simulation package (VASP) [18,19,20]. The interaction between the core and valence electrons is treated via the projector-augmented wave (PAW) method [21,22], and the Perdew–Burke–Ernzerhof generalized gradient approximation (GGA-PBE) is used to describe the exchange and correlation potential [23]. The k-point mesh size with 11 × 11 × 1 and a cutoff energy of 500 eV are employed to optimize the structural parameter. The vacuum thickness of ~20 Å is added to avoid the interlayer reaction between repeated images, and the van der Waals (vdW) interlayer interaction is described using the semi-empirical DFT-D3 method [24]. The energy and force convergences are set as 10^−7^ eV and 0.01 eV/Å, respectively. Because the GGA-PBE usually underestimates the band gaps, the HSE06 method [25,26,27] and the G_0_W_0_ method [28] are both used to calculate band structures. Phonon dispersion curves are computed within a 4 × 4 × 1 supercell approach using the density functional perturbation theory [29] and extracted via the Phonopy code [30,31]. In ab initio molecular dynamics (AIMD) simulations, the initial configuration within the 3 × 3 × 1 supercell is annealed at 300 K in the NVT ensemble, and each AIMD simulation lasts for 8 ps, with a time step of 1 fs. The elastic constants are calculated using the finite different method [29].

To obtain the optical absorption, the 8 × 8 × 1 *k*-point grid is sampled in the G_0_W_0_ + BSE calculations [32,33,34,35]. (1) GGA-PBE calculations with an energy convergence criterion of 1 × 10^−8^ eV are performed; (2) GGA-PBE calculations are restarted to allow for full optical transitions, where ~200 empty bands are added; (3) G_0_W_0_ calculations are carried out to obtain quasi-particle excitations, where an energy cutoff of 150 eV for the response functions, the spectral method, and 72 frequency points are adopted; (4) The Bethe–Salpeter equation is solved, where 22 highest occupied valence bands and 22 lowest unoccupied conduction bands are included.

## 3. Results and Discussion

### 3.1. Structural and Electronic Properties

**Figure 1:**
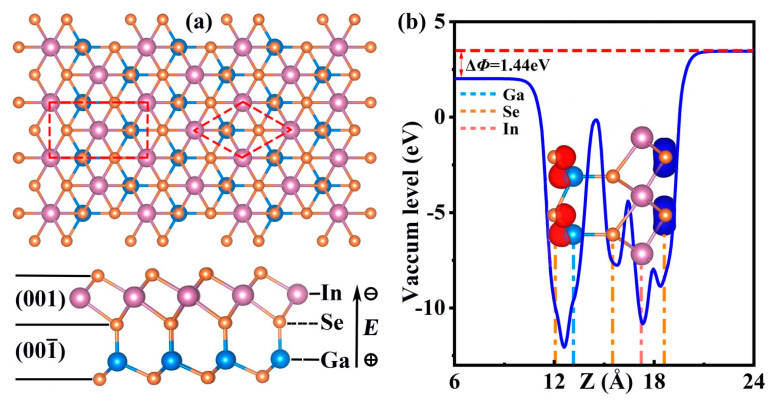
(**a**) The top and side views, where the rhombus denotes the primitive cell and the rectangle represents the orthogonal supercell. (**b**) The vacuum level difference of unstrained single-layer GaInSe_3_, where the inset is the charge distribution of CBM (red) and VBM (blue). The red dashed horizontal lines are auxiliary lines. The solid blue curve shows the change in vacuum level.

Single-layer GaInSe_3_ displays a 2D hexagonal unit cell with an in-plane constant of *a* = 3.95 Å (Figure 1a). Single-layer GaInSe_3_ is stacked by Se-In-Se-Ga-Se atomic layers along the z-direction (Figure 1a), with an effective thickness of ~1 nm (Figure S1). Due to the asymmetric structure, single-layer GaInSe_3_ exhibits an inside electric dipole (field) along the z-direction. The calculated electric dipoles are 9.36, 9.29, 9.26, 9.07, and 9.00 (10^−2^ e·Å) for −2%, −1%, 0%, +1%, and +2% biaxial strains, respectively. Hence, the inside dipole will play an important role in separating the generated holes and electrons. The large inside electric dipole produces a significant vacuum level difference ∆Φ between the bottom Ga-Se layer and the top Se-In-Se layer (Figure 1b and Figure S2 and Table S1).

The stability of unstrained single-layer GaInSe_3_ has been investigated [15], and here we study its stability under biaxial strains. From −2% to 2% biaxial strains, only very small imaginary frequencies appear near the Γ point, and they could be eliminated gradually by enlarging the supercell [36], expressing good dynamic stability (Figure S3). Single-layer GaInSe_3_ under biaxial strains from −2 to +2% exhibits free energy fluctuating around −160 eV and maintains the integrity of structural frames throughout the simulation process (Figures S4 and S5), proving thermal stability at 300 K. The theoretical elastic constants *C*_ij_ (Table S2) satisfy the Born–Huang mechanical stability criterion of lattice dynamics [37,38,39]: *C*_11_ > 0 and *C*_11_ − *C*_12_ > 0 suggesting mechanical stability. What is more, the total energy under the considered strain is very close to that at the ground state (Figure S6). Therefore, the dynamic stability, mechanical stability, and thermal stability under the considered biaxial strains have shown that single-layer GaInSe_3_ can be realized in experiments.

**Figure 2:**
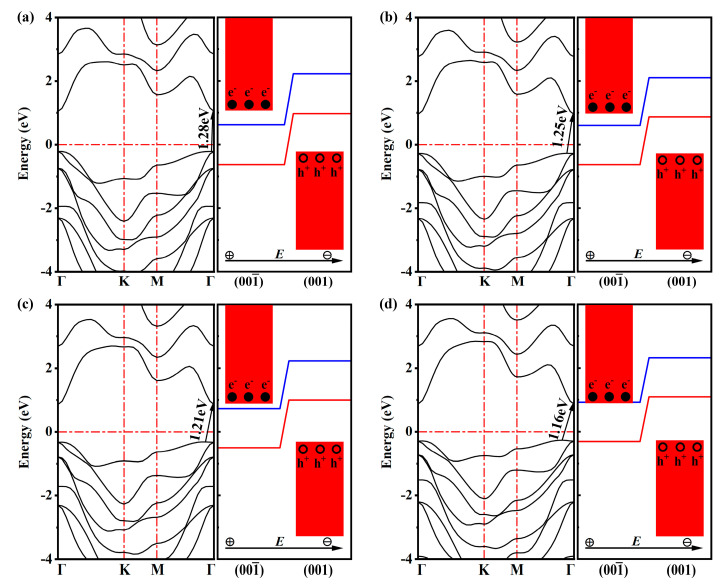
The electronic band structure (**Left**) and water-splitting property (**Right**) of single-layer GaInSe_3_ under biaxial strains of (**a**) −2%, (**b**) −1%, (**c**) 0%, and (**d**) +1% obtained via HSE06. The upper and lower red columns denote the CBM and the VBM, respectively. The blue and red lines represent the redox potentials of H^+^/H_2_ and H_2_O/O_2_, respectively.

A quasi-direct energy gap appears for single-layer GaInSe_3_ under biaxial strains from −2% to +2%. Bandgaps are 1.28 eV for −2%, 1.25 eV for −1%, 1.21 eV for 0%, 1.16 eV for +1%, and 1.11 eV for +2% strain (Figure 2a–d and Figure S7). Notably, the moderate bandgaps of single-layer GaInSe_3_ are very helpful for visible light absorption. Furthermore, due to the vacuum level difference between the top and bottom surfaces, the VBM of unstrained single-layer GaInSe_3_ is located at the top Se-In-Se trilayer—more precisely, at the highest Se atomic layer. Its CBM is mainly seated in the bottom Ga-Se bilayer (Figure 1b). Thus, the CBM and VBM are spatially separated, thus decreasing the electron–hole binding rate and improving the photocatalytic performance. The separated CBM and VBM also appear in single-layer GaInSe_3_ under biaxial strains from −2% to 2% (Figure S8).

In addition, the spin–orbital coupling (SOC) has little influence on the electronic property of single-layer GaInSe_3_ (Figure S9). The bandgaps at the G_0_W_0_ level are presented in Figure S10. Since the bandgaps of 2D materials are underestimated by PBE and overestimated by G_0_W_0_ [16], only the HSE06 bandgaps are focused on herein.

### 3.2. Band Alignment for Water-Splitting

As for water-splitting by sunlight, the most important prerequisite is that the aligned CBM level is higher than the H^+^/H_2_ reduction potential ($E_{H^{+}/H_{2}}$), while the aligned VBM level is below the O_2_/H_2_O oxidation potential ($E_{O_{2}/H_{2}O}$). Herein, the band edges are aligned using the method proposed by Toroker et al. [40] and revised by Yang et al. [10]. Considering the charge location of CBM and VBM (Figure 1b), the hydrogen evolution reactions (HERs) of single-layer GaInSe_3_ take place on the Ga atomic layer, and the oxygen evolution reactions (OERs) mainly occur on the highest Se atomic layer. From −2% to +2% biaxial strains, the band edges of single-layer GaInSe_3_ are favorable for OERs (Figure 2a–d and Figure S7). However, the band edges under +1% and +2% tensile biaxial strains are unfavorable for HERs. As a result, single-layer GaInSe_3_ under biaxial strains from −2% to 0% can accomplish both HERs and OERs.

The overpotentials, including χ(H_2_) and χ(O_2_), which, respectively, scale the water-splitting abilities of HERs and OERs, are further studied (Table 1). The χ(H_2_) (χ(O_2_)) is the potential difference between the aligned CBM (VBM) level and the H^+^/H_2_ (O_2_/H_2_O) potential. Under biaxial strains of −2% and −1%, the χ(H_2_) values are larger than 0.2 eV, and the χ(O_2_) values are larger than 0.6 eV, indicating good HER and OER ability [10]. In addition, the potential energies of photogenerated carriers ($U_{e}$ and $U_{h}$) are also calculated to reveal the HER and OER abilities [41,42], also suggesting good HER and OER ability for single-layer GaInSe_3_ at −2% and −1% biaxial strain.

### 3.3. Optical Absorption and Exciton Binding Energy

**Figure 3:**
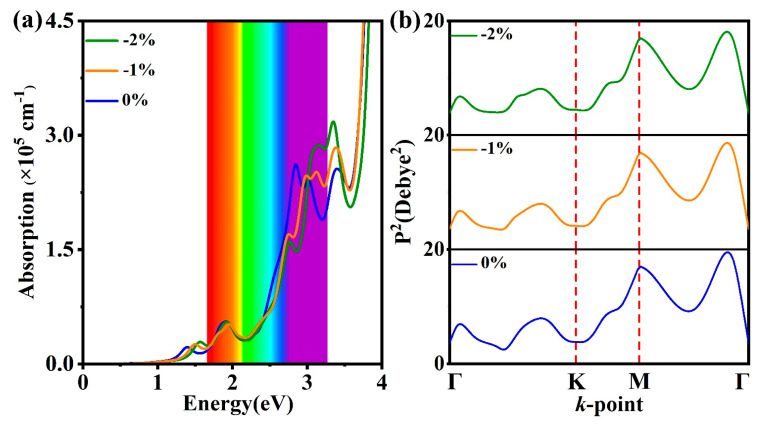
(**a**) Frequency-dependent absorption coefficients of single-layer GaInSe_3_ under biaxial strains of −2%, −1%, and 0% by G_0_W_0_-BSE. (**b**) Transition dipole moment of single-layer GaInSe_3_ under biaxial strains of −2%, −1%, and 0% by HSE06.

For water-splitting, the photocatalysts should absorb as much as solar energy. Herein, the G_0_W_0_-BSE method is applied to study the absorption coefficient [32,33,34,35,43]. Due to the existence of a vacuum, the effective thickness is considered [44]. Calculations reveal that the first absorption peaks of GaInSe_3_ under biaxial strains of −2%, −1%, and 0% are located at 1.57, 1.50, and 1.39 eV, respectively (Figure 3a). From −2% to 0% biaxial strains, single-layer GaInSe_3_ exhibits multiple visible optical absorption peaks, and the maximum optical absorption reaches up to ~ 3 × 10^5^ cm^−1^, apparently stronger than that of other typical 2D photocatalysts such In_2_Se_3_ [45]. It is not difficult to find that the maximum optical absorption is increasing with the decreasing compressive biaxial strain. Moreover, the optical absorption spectra are red-shifted with the decreasing compressive biaxial strain. On the other hand, the square transition dipole matrix elements (*P*^2^) between the top VB and the bottom CB are calculated via HSE06 [46]. From −2% to 0% biaxial strains, the maximum *P*^2^ appears near the Γ point and approaches 20 Debye^2^ (Figure 3b). The allowed optical transition further promises infrared and visible light absorption.

Water-splitting reactions require separated photogenerated electrons and holes. The exciton binding energy is an important parameter by which to measure the separation efficiency of charge carriers. Considering the quasi-direct bandgap of single-layer GaInSe_3_, the exciton binding energy $E_{b}$ is the energy difference between the first optical bandgaps (Figure 3a) and quasi-direct bandgaps (Figure S10). The quasi-direct bandgaps of single-layer GaInSe_3_ under biaxial strains from −2%, −1%, and 0% are 2.03, 2.07, and 2.13 eV, respectively. The corresponding $E_{b}$ values are 0.46, 0.57, and 0.74 eV, and lower than those of 2D photocatalysts, e.g., In_2_Se_3_ (0.69 eV) [10] and WSSe (0.82 eV) [17]. First, the relatively smaller exciton binding energies could be attributed to the separated CBM and VBM. Secondly, the inside electric field could play a big role in reducing the exciton binding energies.

### 3.4. Transport Mobility

The recombination rate of charge carriers is further investigated by including carrier mobility. Expressly, higher mobility means a lower recombination possibility. Herein, The carrier mobility is calculated using the deformation potential (DP) theory [47,48]:

$$\mu_{2D}=\frac{e\hbar^{3}C_{2D}}{k_{B}Tm^{*}m_{d}E_{d}^{2}}.$$

Here, *e* is the electron charge, ℏ is the reduced Planck constant, *C*_2*D*_ is the elastic constant, $k_{B}$ is the Boltzmann constant, and *T* is the room temperature (300 K). $m^{*}$ is the effective mass and can be written as $\frac{1}{m^{*}}=\frac{1}{\hbar^{2}}\left|\frac{\partial^{2}E\left(k\right)}{\partial k^{2}}\right|$. The average effective mass $m_{d}$ is expressed as $m_{d}=\sqrt{m_{x}^{*}m_{y}^{*}}$. The symbol $E_{d}$ represents the deformation potential constant. The fittings for effective masses $m^{*}$ and deformation potential constants $E_{d}$ are listed in Figures S11–S16. Herein, the $E_{d}$ values are obtained with the consideration of VBM and CBM distribution (Figure 1b). Specifically, the $E_{d}$ value of generated holes is taken as the slope of the linear fitting between ($E_{VBM}-\Phi_{top}$) and strain *ε*. *E_d_* of generated electrons is taken as the slope of the linear fitting between ($E_{VBM}-\Phi_{bottom}$) and ε. The calculated effective masses $m^{*}$, elastic constants $C_{2D}$, and deformation potential constants $E_{d}$ are summarized in Table 2.

For unstrained single-layer GaInSe_3_, the electron effective masses (0.15 *m_e_*) and elastic constants (78.36 N/m) along the *x* and *y* directions are both isotropic. Due to the small electron-effective masses, a relatively large mobility (~1250 cm^2^∙V^−1^∙s^−1^) of photogenerated electrons is obtained. For comparison, recently discovered 2D photocatalysts display electron mobility: 1049 cm^2^∙V^−1^∙s^−1^ for GeS [49] and 601 cm^2^∙V^−1^∙s^−1^ for CoGeSe_3_ [50]. Moreover, apparent hole mobility anisotropy between the *x* and *y* directions appears owing to various effective masses and deformation potential constants. Another prominent characteristic is that the generated electrons run much faster than the generated holes, which promises an effective separation of photogenerated electrons and holes. Under −2% and −1% biaxial strains, effective separation of photogenerated carriers inside single-layer GaInSe_3_ still exists, and then ensures the occurrence of photocatalytic reactions.

### 3.5. OER and HER

Although single-layer GaInSe_3_ exhibits a suitable band edge for redox reaction under biaxial strains from −2% to 0%, it is necessary to calculate the Gibb free energy in OER and HER. Combined with the analysis of the charge density of VBM and CBM, the HER will take place on the bottom Ga-Se bilayer, and the OER will take place on the highest Se atomic layer. The OER is divided into four steps, as follows in Equations (1)–(4) [2,51]:

(1) $$*+H_{2}O\to {OH}^{*}+H^{+}+e^{-}$$

(2) $${OH}^{*}\to O^{*}+H^{+}+e^{-}$$

(3) $$O^{*}+H_{2}O\to {OOH}^{*}+H^{+}+e^{-}$$

(4) $${OOH}^{*}\to *+O_{2}+H^{+}+e^{-}.$$

The two steps of HER are given by Equations (5) and (6) [2,51]:

(5) $$*+H^{+}+e^{-}\to H^{*}$$

(6) $$H^{*}+H^{+}+e^{-}\to *+H_{2},$$

where * indicates the adsorbed material (single-layer GaInSe_3_), and ${OH}^{*}$, $O^{*}$, ${OOH}^{*}$, and $H^{*}$ represent the adsorbed intermediates. For all the calculations, spin polarization is taken into account. By considering the zero-point energy and entropy corrections, the expression of ∆G can be written as $∆G={∆E}_{ads}+{∆E}_{ZPE}\;–\;T∆S$, where ${∆E}_{ads}$ is the adsorption energy, and ${∆E}_{ZPE}$ and ∆S are the difference of zero-point energy and entropy difference between the adsorbed state and the gas phase, respectively. *T* represents the room temperature of 300 *K*.

For each reaction of oxidation generation, ΔG can be written as follows in Equations (7)–(10) [2,51]:

(7) $$∆G_{1}=G_{{OH}^{*}}+\frac{1}{2}G_{H_{2}}-G^{*}-G_{H_{2}O}+∆G_{U}-∆G_{pH}$$

(8) $$\Delta G_{2}=G_{O^{*}}+\frac{1}{2}G_{H_{2}}-G_{{OH}^{*}}+∆G_{U}-∆G_{pH}$$

(9) $$∆G_{3}=G_{{OOH}^{*}}+\frac{1}{2}G_{H_{2}}-G_{O^{*}}-G_{H_{2}O}+∆G_{U}-∆G_{pH}$$

(10) $$∆G_{4}=G^{*}+\frac{1}{2}G_{H_{2}}+G_{O_{2}}-G_{{OOH}^{*}}+∆G_{U}-∆G_{pH},$$

where $∆G_{U}\;(∆G_{U}=-eU)$ denotes extra potential bias provided by an electron in the electrode, and U is the electrode potential relative to the standard hydrogen electrode (SHE). $∆G_{pH}(∆G_{pH}=k_{B}T\times ln10\times pH)$ represents the free energy contributed in different pH.

**Figure 4:**
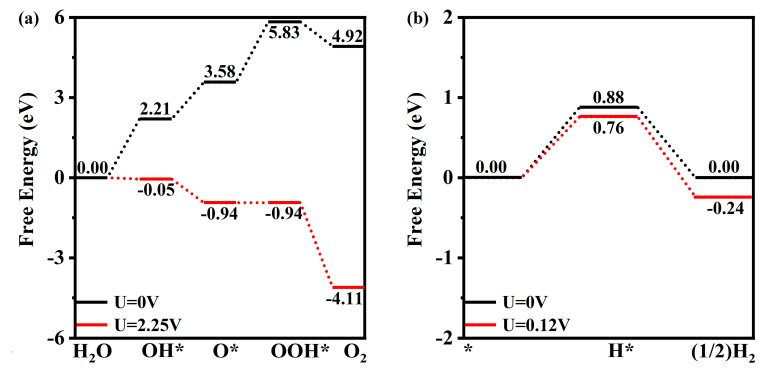
(**a**) The Gibbs free energy change of OER on the Se atomic plane; (**b**) that of HER on the bottom Ga atomic layer of unstrained single-layer GaInSe_3_ at pH = 0. The * indicates the adsorbed material.

Because of the required large computational resources, only the OER and HER of unstrained single-layer GaInSe_3_ are studied. The optimized configurations of the ${OH}^{*}$, $O^{*}$, and ${OOH}^{*}$ intermediates are given (Figure S17). The formation of ${OH}^{*}$, $O^{*}$, and ${OOH}^{*}$ are all endothermic in the absence of solar light (Figure 4a). The third step is a rate-limiting step involving the ${OOH}^{*}$ formation and has a Gibbs free energy change of 2.25 eV. Therefore, the minimum external potential for OER converted into exothermic heat is 2.25 V. As shown in Table 1, for unstrained single-layer GaInSe_3_, the external electric potential supplied by the photogenerated hole is 2.50 V $(U_{h}^{,}=U_{h}/e$), indicating that OER can proceed smoothly without a co-catalyst.

On the other hand, the Gibbs free energy profile of HER is displayed in Figure 4b. The optimized configurations of the $H^{*}$ intermediate are given (Figure S18), where the HER active site is the Ga atomic layer. In the absence of light irradiation, the first and second steps are endothermic and exothermic with ΔG = 0.88 eV, respectively. Therefore, the minimum external potential for a successful HER is 0.88 V. However, the external electric potential of unstrained single-layer GaInSe_3_ (Table 1) supplied by the photogenerated electrons is 0.12 V. In this condition, the first step involving the H* formation is also endothermic, with ΔG = 0.76 eV, indicating that HER cannot proceed successfully without a cocatalyst. Fortunately, in previous experiments, the HER energy barrier could be lowered to less than 0.1 eV [52].

### 3.6. Solar-to-Hydrogen Efficiency

Here, we investigate the solar conversion efficiency of single-layer GaInSe_3_ in water-spitting reactions. Generally, the $\eta_{STH}$ value is obtained by timing the light absorption efficiency ($\eta_{abs})$ and the carrier utilization efficiency ($\eta_{cu}$). The $\eta_{abs}$ value is calculated via Equation (11):

(11) $$\eta_{abs}=\frac{\int_{E_{g}}^{\infty }P\left(\hbar w\right)d(\hbar w)}{\int_{0}^{\infty }P\left(\hbar w\right)d(\hbar hw)},$$

where $P\left(\hbar w\right)$ is the AM1.5G solar energy flux at the photon energy ℏw, and $E_{g}$ is the bandgap by HSE06 (Figure 2 and Figure S6). Due to the narrower bandgap, the $\eta_{abs}$ values of single-layer GaInSe_3_ under biaxial strains from −2% to 0% exceed 70%.

The $\eta_{cu}$ is calculated via Equation (12):

(12) $$\eta_{cu}=\frac{∆G\int_{E}^{\infty }\frac{P\left(\hbar w\right)}{\hbar w}d(\hbar w)}{\int_{E_{g}}^{\infty }P\left(\hbar w\right)d(\hbar w)},$$

where ∆G=1.23 eV is the potential difference for photocatalytic water-splitting. The symbol E is the actual energy barrier for photocatalytic water-splitting and is determined via Equation (13):

(13) $$E=\left\{\begin{array}{c} E_{g},\;(\chi (H_{2})\geq 0.2,\chi (O_{2})\geq 0.60)\\ E_{g}+0.2-\chi (H_{2}),\;(\chi \left(H_{2}\right)<0.2,\chi (O_{2})\geq 0.60)\\ E_{g}+0.6-\chi (O_{2}),\;(\chi \left(H_{2}\right)\geq 0.2,\chi \left(O_{2}\right)<0.60)\\ E_{g}+0.8-\chi (H_{2})-\chi (O_{2}),\;(\chi \left(H_{2}\right)<0.2,\chi \left(O_{2}\right)<0.60) \end{array}\right..$$

The overpotentials of χ(H_2_) and χ(O_2_) values are listed in Table 1. Furthermore, the $\eta_{cu}$ values from −2% to 0% are found to surpass 70%. Herein, the STH efficiencies ($\eta_{STH})$ under biaxial strains of −2%, −1%, and 0% via Equation (14) are 44.18%, 45.85%, and 44.00%, respectively.

(14) $$\eta_{STH}=\eta_{abs}\times \eta_{cu}$$

The $\eta_{STH}$ is further corrected by the inside electric field via Equation (15):

(15) $$\eta_{STH}^{,}=\eta_{STH}\times \frac{\int_{0}^{\infty }P\left(\hbar w\right)d(\hbar w)}{\int_{0}^{\infty }P\left(\hbar w\right)d\left(\hbar w\right)+∆\Phi \int_{E_{g}}^{\infty }\frac{P\left(\hbar w\right)}{\hbar w}d(\hbar w)},$$

where ∆Φ is the potential difference between the vacuum level at the top and bottom surfaces (Table 1). Very excitingly, the corrected STH efficiency ($\eta_{STH}^{,}$) of single-layer GaInSe_3_ under biaxial strains of −2%, −1%, and 0% reaches 31.20%, 32.50%, and 31.29%, respectively, far surpassing the commercial standard (10%) [10] and showing glorious prospects for commercial application (Table 3). It should be pointed out that we assume the external quantum efficiency of 100% for the overall water-splitting reaction; that is, the obtained STH efficiency is a theoretical limitation. Lower SHT efficiency was obtained in the experiments. Specifically, the low $U_{e}$ of single-layer GaInSe_3_ under zero strain suggests possible photo corrosion, which will reduce the STH efficiency.

## 4. Conclusions

The solar water-splitting properties of single-layer GaInSe_3_ have been studied. Under biaxial strains from −2% to +2%, single-layer GaInSe_3_ exhibits stable structures and moderate bandgaps from 1.11 to 1.28 eV. Under biaxial strains from −2% to 0%, single-layer GaInSe_3_ holds suitable band edges for photocatalytic water-splitting at pH = 0, and the strong visible optical absorption is up to ~3 × 10^5^ cm^−1^. Meanwhile, the charge carriers can be effectively separated due to the strong inside electric field and high electron mobility. More attractively, the solar energy conversion efficiency surpasses 30%. The OER of unstrained single-layer GaInSe_3_ can proceed without a co-catalyst. Therefore, single-layer GaInSe_3_ is a promising photo-catalyst material for water-splitting.

## Figures and Tables

**Table 1:** The overpotentials of HER (χ(H_2_), $U_{e}$) and OER (χ(O_2_), $U_{h}$) (eV) via HSE06 at pH = 0.

	χ(H_2_)	χ(O_2_)	$U_{e}$	$U_{h}$
−2%	0.46	1.03	0.46	2.26
−1%	0.30	1.17	0.30	2.40
0%	0.12	1.27	0.12	2.50

**Table 2:** Elastic modulus *C*_2*D*_ (N·m^−1^), effective mass $m^{*}$ (*m_e_*), deformation potential *E_d_* (eV), and carrier mobility μ (cm^2^∙V^−1^∙s^−1^) along the *x* and *y* directions of single-layer GaInSe_3_ under biaxial strains from −2% to 0%.

Stain	Direction	Species	$m^{*}$	$C_{2D}$	$E_{d}$	μ
−2%	*x*	Electron	0.16	85.61	4.66	3455.11
		Hole	0.40	85.61	5.08	95.86
	*y*	Electron	0.16	85.61	6.22	2901.05
		Hole	3.71	85.61	6.74	8.85
−1%	*x*	Electron	0.15	82.13	7.79	1205.60
		Hole	0.35	82.13	3.75	342.64
	*y*	Electron	0.15	82.13	7.70	1232.79
		Hole	2.96	82.13	5.38	19.95
0%	*x*	Electron	0.15	78.36	7.40	1281.00
		Hole	0.34	78.36	2.64	748.44
	y	Electron	0.15	78.36	7.58	1223.19
		Hole	2.58	78.36	5.71	21.11

**Table 3:** The energy conversion efficiency of light absorption ($\eta_{abs}$), carrier utilization ($\eta_{cu}$), solar-to-hydrogen (STH) ($\eta_{STH}$), and corrected STH ($\eta_{STH}^{,}$) of single-layer GaInSe_3_ under biaxial strains of −2%, −1%, and 0%.

Species	Strain	$\eta_{abs}(\%)$	$\eta_{cu}(\%)$	$\eta_{STH}(\%)$	$\eta_{STH}^{,}(\%)$
pH = 0	−2%	71.02	79.63	44.18	31.20
	−1%	72.74	78.80	45.85	32.50
	0%	75.05	70.93	44.00	31.29

## Data Availability

Not applicable.

## Supplementary Materials

The following supporting information can be downloaded at https://www.mdpi.com/article/10.3390/molecules28196858/s1. Figure S1: Effective thickness; Figure S2 and Table S1: Vacuum level difference; Figure S3: Phonon dispersion; Figures S4 and S5:Thermal stability; Figure S6: Total energy under the considered strain; Figure S7: HSE06 band structure and the band alignment under the −2% biaxial strain; Figure S8: Spatial distribution of CB and VB; Figure S9: PBE band with and without SOC; Figure S10: G_0_W_0_ band; Figures S11–S16: Quadratic and linear fittings for transport mobility; Figure S17: Configurations of OH*, O* and OOH*; Figure S18: Configurations of H*; Table S2: Elastic constants; Table S3: Free energy related physical quantities.

## Sample Availability

Samples of the compounds are available from the authors.
