# Supplementary material for: Single-Layer GaInSe3: Promising Water-Splitting Photocatalyst with Solar Conversion Efficiency over 30% from Theoretical Calculations
Source: Molecules. 2023 Sep 28;28(19):6858. doi: 10.3390/molecules28196858 (PMC10574629; doi:10.3390/molecules28196858)
Supplement: Supplementary file 1 [file molecules-28-06858-s001.zip › molecules-2573189-supplementary.pdf]

molecules

MDPI

Article

Single-Layer $GaInSe_3$: Promising Water-Splitting Photocatalyst with Solar Conversion Efficiency Over 30% from Theoretical Calculations

Li-Li Liu [1,2,*], Ru-Fei Tang [1], De-Fen Li [1], Ming-Xia Tang [1], Bing-Zhong Mu [1], Zheng-Quan Hu [3], Shi-Fa Wang [3], Yu-Feng Wen [4,*] and Xiao-Zhi Wu [2,*]

[1] College of Teacher Education, Chongqing Three Gorges University, Chongqing 404100, China; TangRFsanxu@163.com (R.-F.T.); LiDFsanxu@163.com (D.-F.L.); tangmxsanxu@163.com (M.-X.T.); m15213132982@163.com (B.-Z.M.)
[2] Institute for Structure and Function, Chongqing University, Chongqing 401331, China; xiaozhiwu@cqu.edu.cn
[3] College of Electronic and Information Engineering, Chongqing Three Gorges University, Chongqing 404100, China; hzq250314@163.com (Z.-Q.H.); wangshifa2006@yeah.net (S.-F.W.)
[4] School of Mathematical Sciences and Physics, Jinggangshan University, Ji'an 343009, China
* Correspondence: liulili0612@163.com (L.-L.L.); wenyufeng@jgsu.edu.cn (Y.-F.W.)

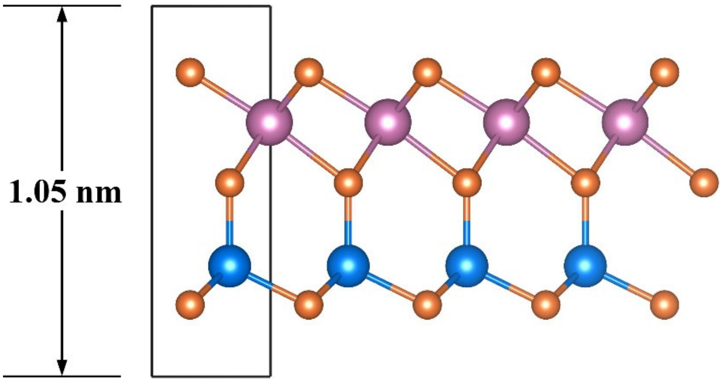

Figure S1. The assumed bulk $GaInSe_3$ crystal containing one $GaInSe_3$ layer.

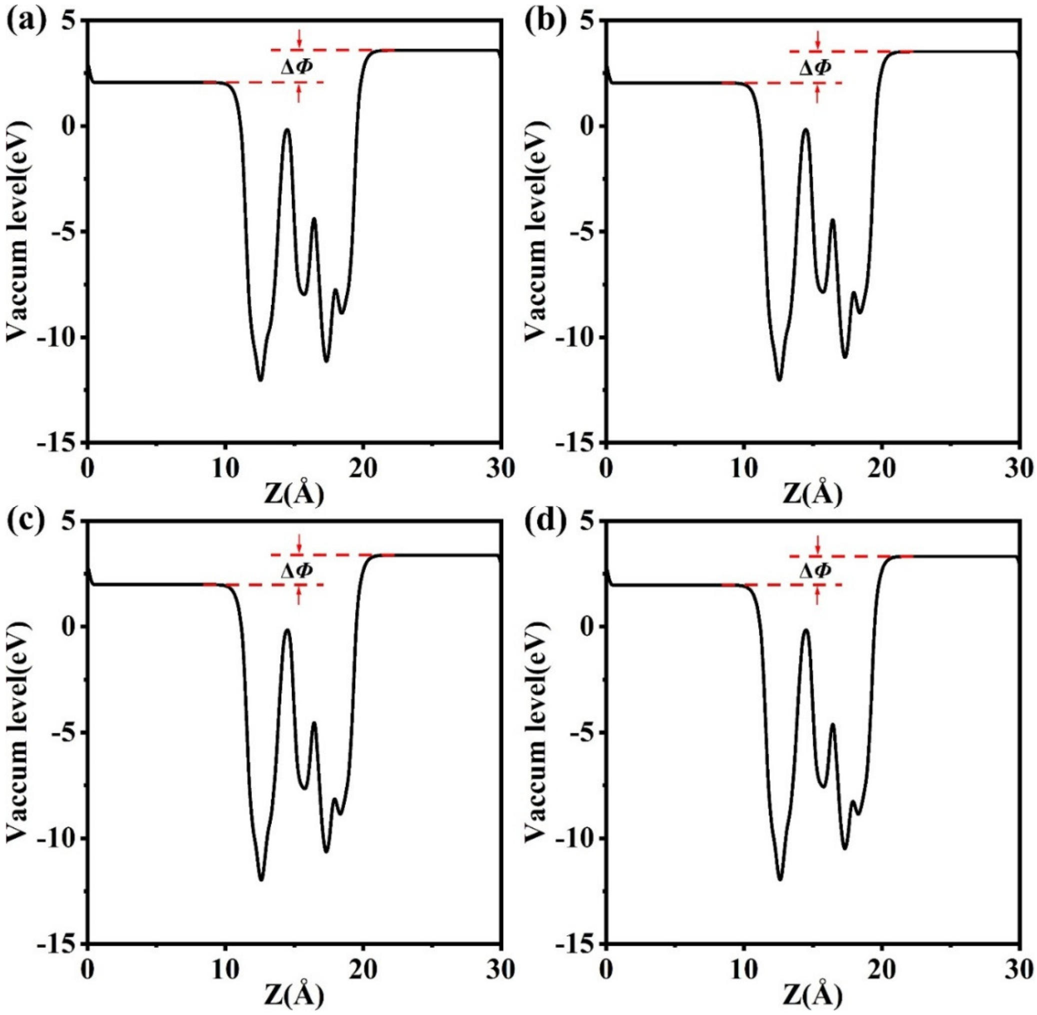

Figure S2. The vacuum level difference of single-layer $GaInSe_3$ under the biaxial strains of (**a**) -2%, (**b**) -1%, (**c**) +1%, and (**d**) +2%.

The vacuum level differences of single-layer $GaInSe_3$ under the biaxial strains from -2% to +2% between top and bottom surfaces are listed in Table S1.

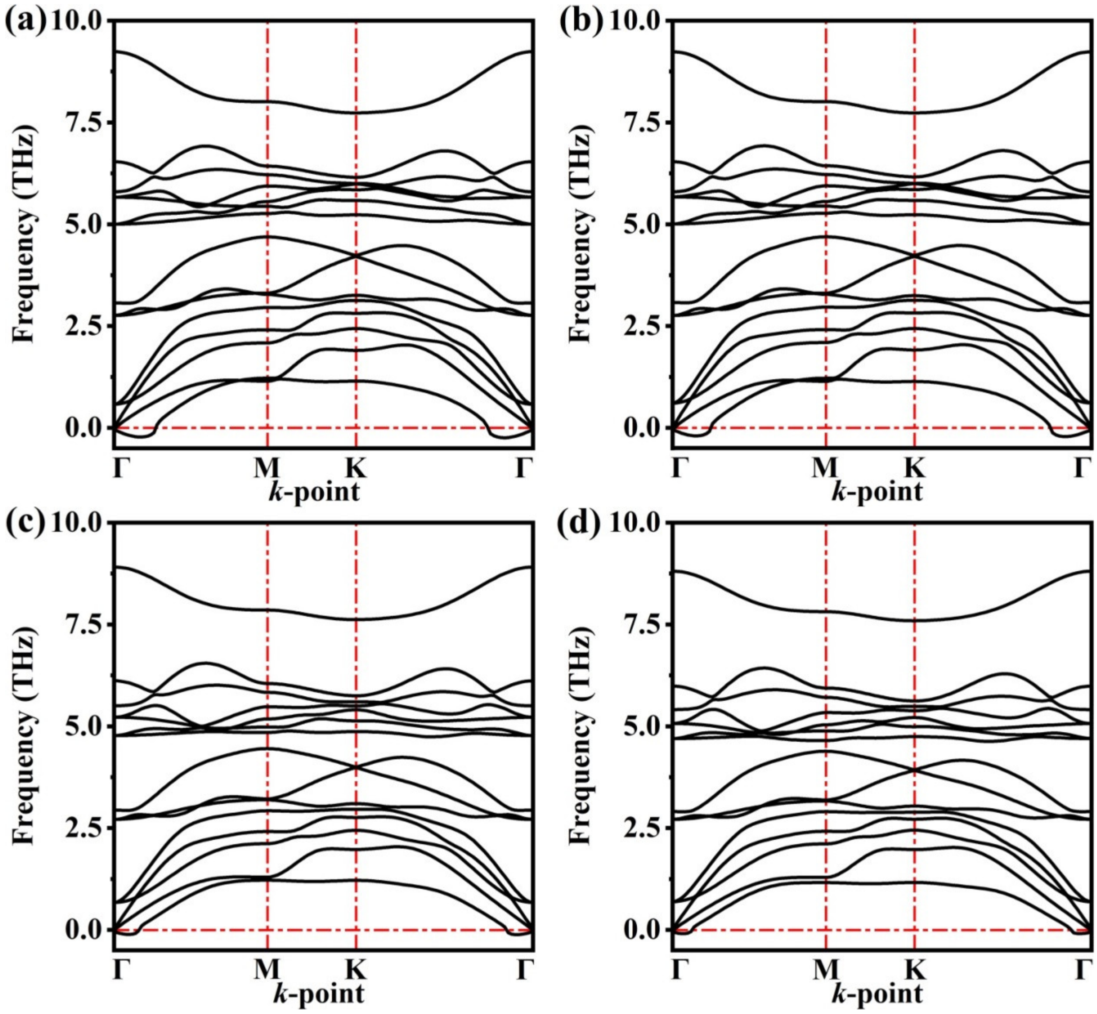

Figure S3. The phonon dispersion curve of single-layer $GaInSe_3$ under the biaxial strains of (**a**) -2%, (**b**) -1%, (**c**) +1%, and (**d**) +2%.

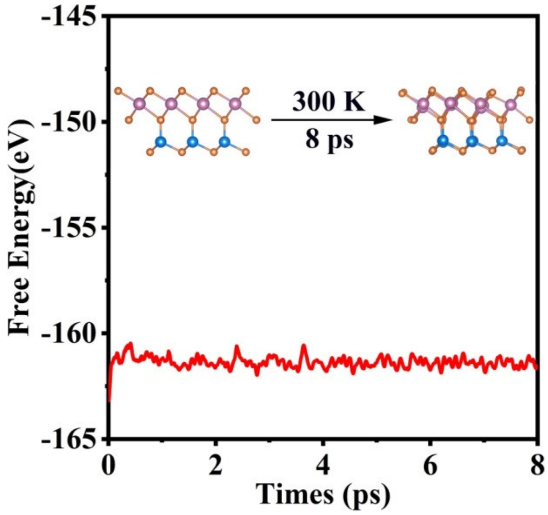

Figure S4. The thermal stability of unstrained single-layer $GaInSe_3$.

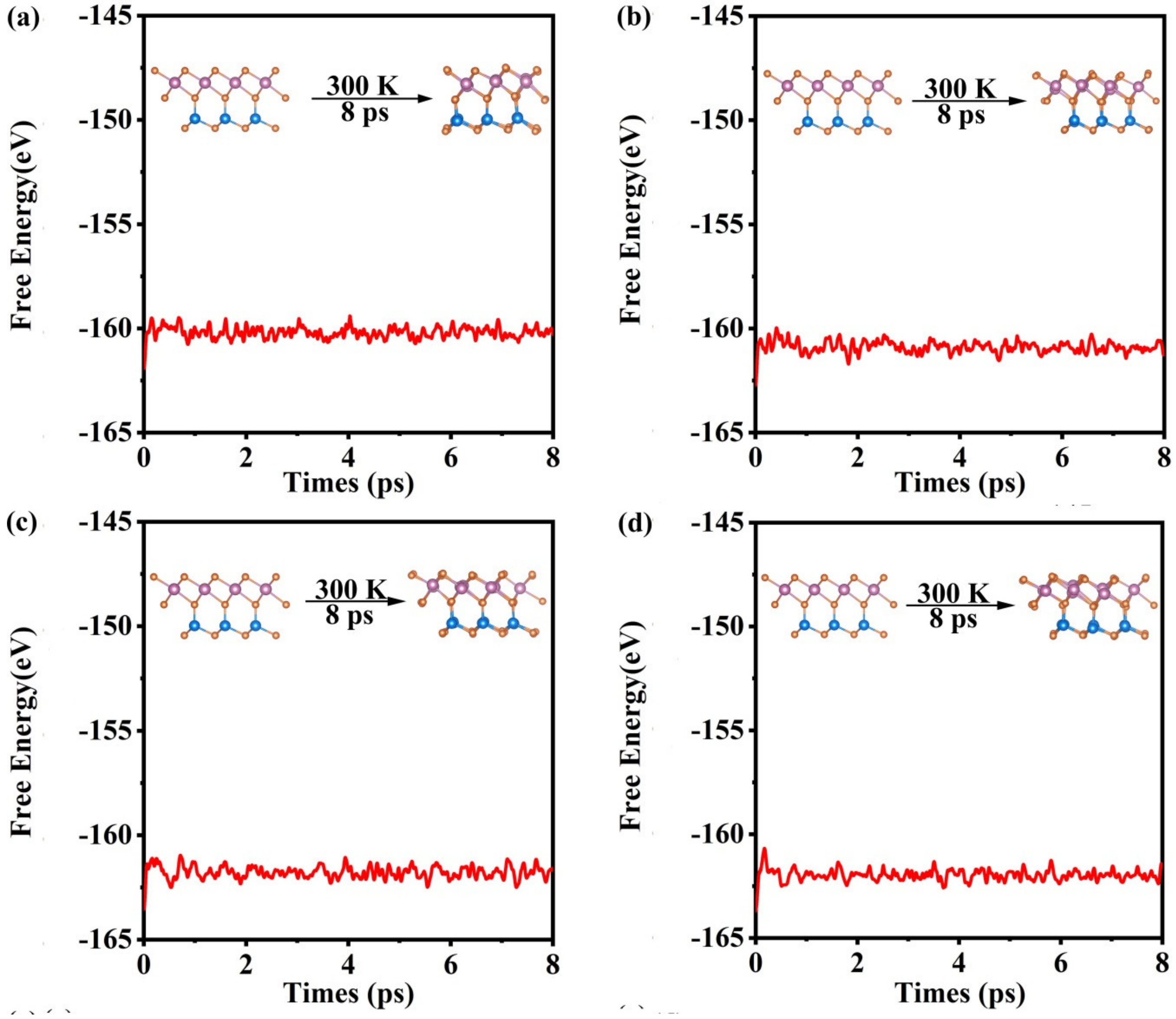

Figure S5. The snapshots of single-layer $GaInSe_3$ under the biaxial strains of (**a**) -2%, (**b**) -1%, (**c**) +1%, (**d**) +2% under before and after AIMD simulations at 300 K.

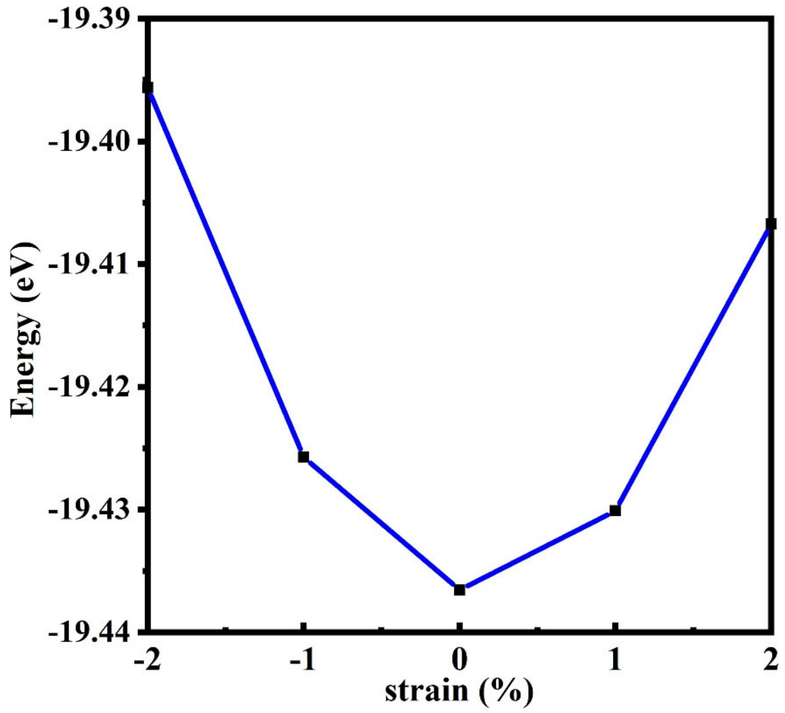

Figure S6. The total energy under the considered strains.

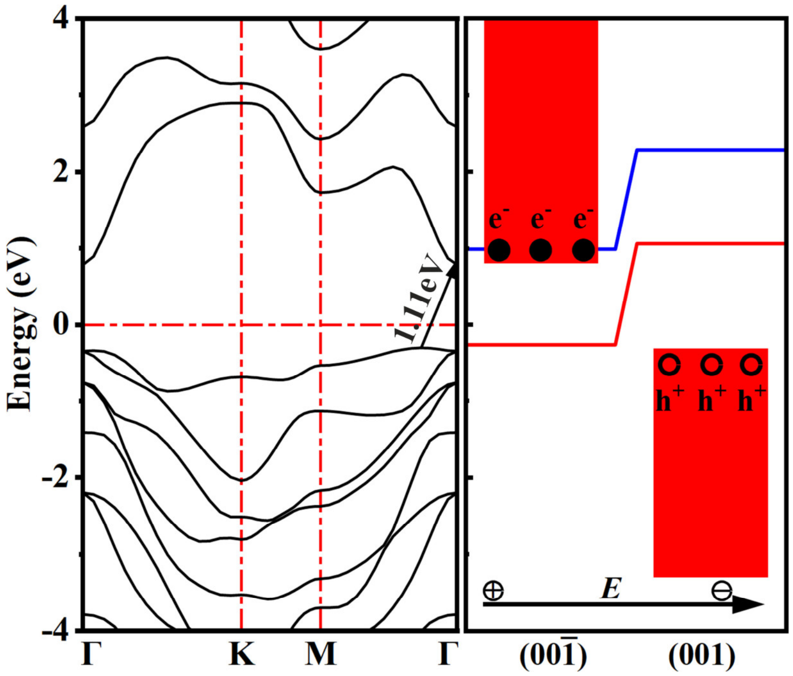

Figure S7. The band structure and the band alignment of single-layer $GaInSe_3$ under the biaxial strain of +2% calculated using HSE06.

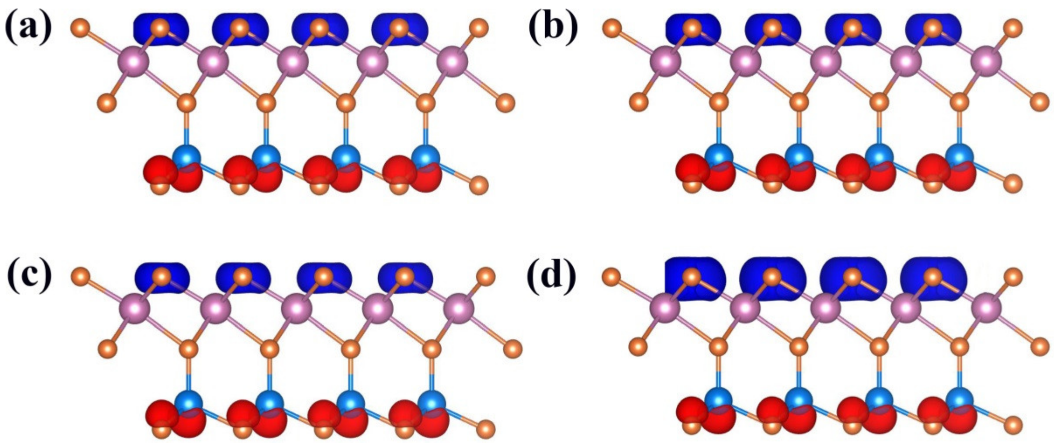

Figure S8. The spatial distribution of CBM (red) and VBM (blue) of single-layer $GaInSe_3$ under the biaxial strains of (**a**) -2%, (**b**) -1%, (**c**) +1%, and +2%.

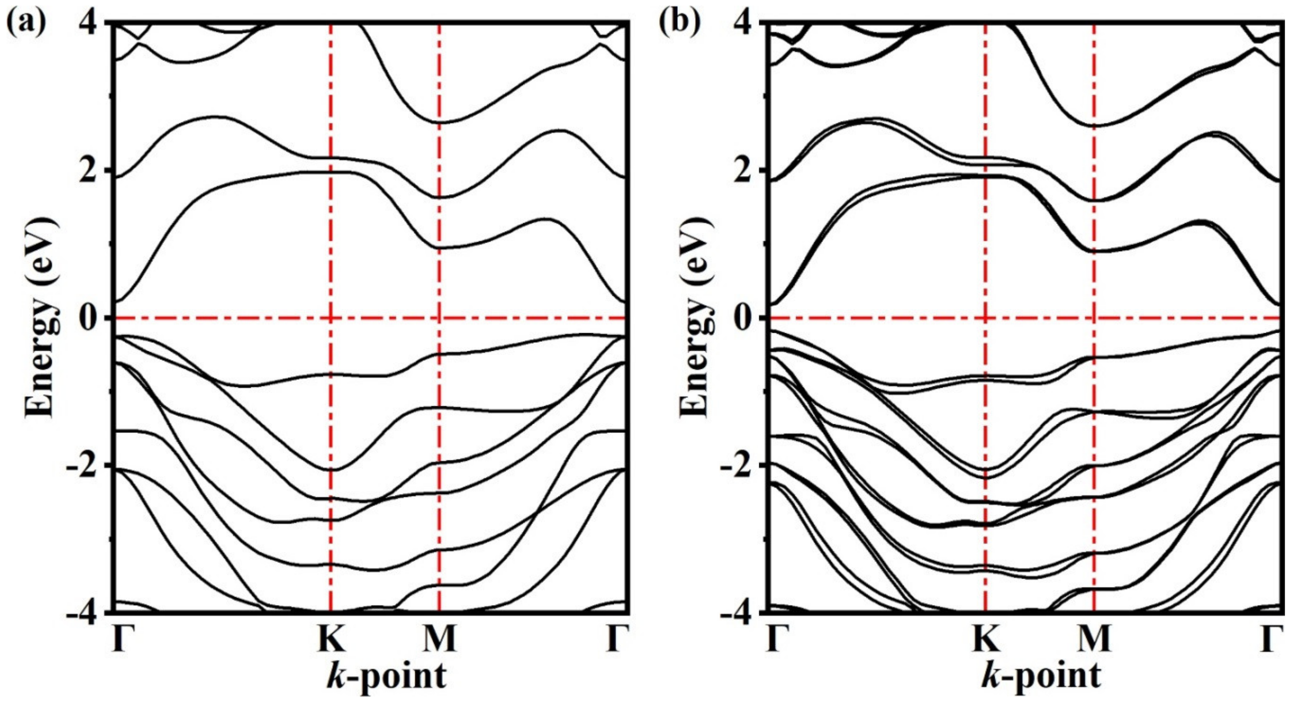

Figure S9. The band structures of unstrained single-layer $GaInSe_3$ at the (a) PBE and (b) PBE+SOC level.

As usual cases, one band is splitting into two bands when the SOC is included. Notably, the band splitting is not apparent, and especially it is negligible for the top valence band and the bottom conduction band. Thus, the SOC can be ignored when exploring the electronic structure of single-layer $GaInSe_3$.

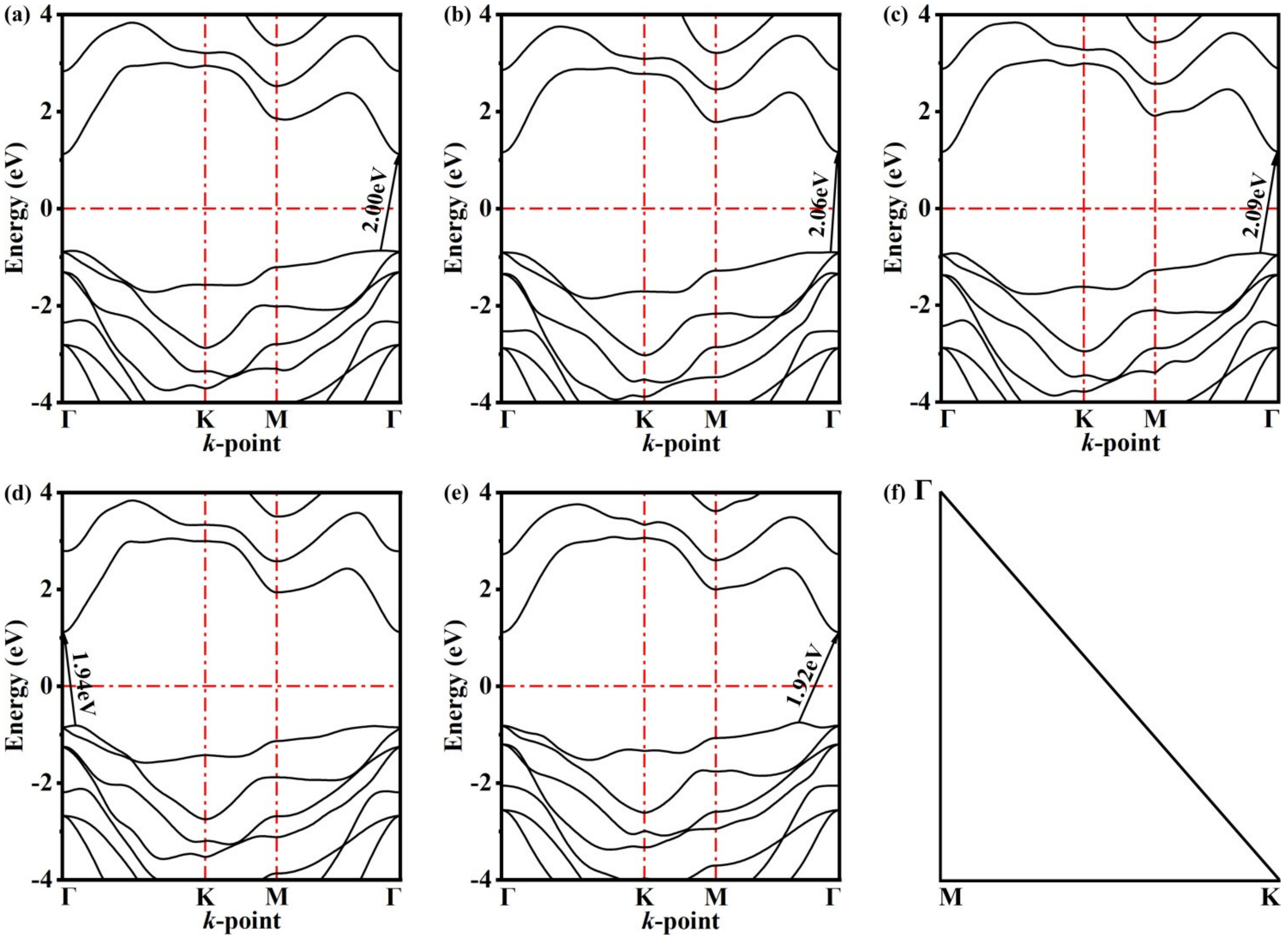

Figure S10. The band structures of single-layer $GaInSe_3$ under the biaxial strains of (**a**) -2%, (**b**) -1%, (**c**) 0%, (**d**) +1%, (**e**) +2% calculated at the G_0W_0, and (**e**) the 2D Brillouin Zone.

The bandgaps could be overestimated due to the inclusion of excitonic effects. Hence, the G_0W_0 bands are not discussed.

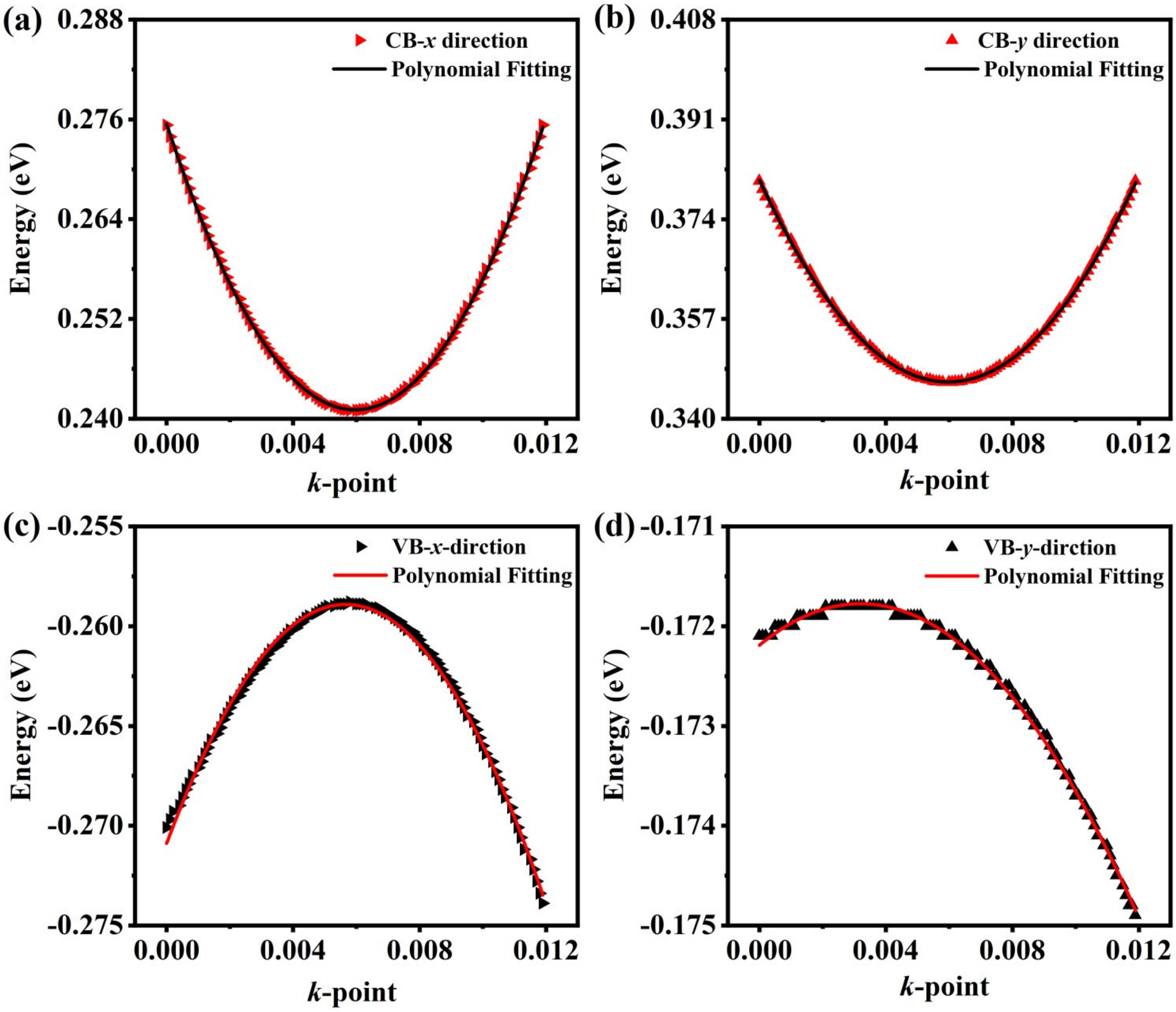

Figure S11. The quadratic fitting between the energy near the CBM (VBM) and k-point along the x and y directions under the biaxial strain of -2%.

According to the DP theory, the effective mass m^* is obtained by the quadratic fitting between the energy near the CBM (VBM) and the k-point.

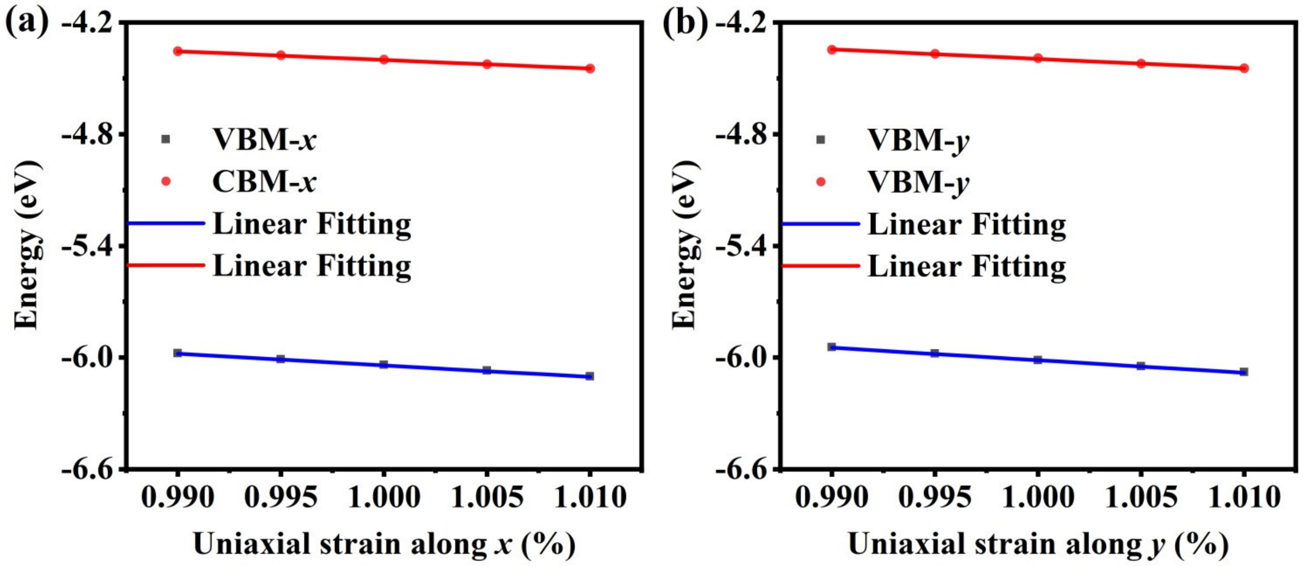

Figure S12. The linear fitting for the deformation potential constants along the x and y directions of single-layer $GaInSe_3$ under the biaxial strain of -2%.

According to the DP theory, the deformation potential constant is calculated based on the linear fitting between the energy of CBM (VBM) and the uniaxial strain.

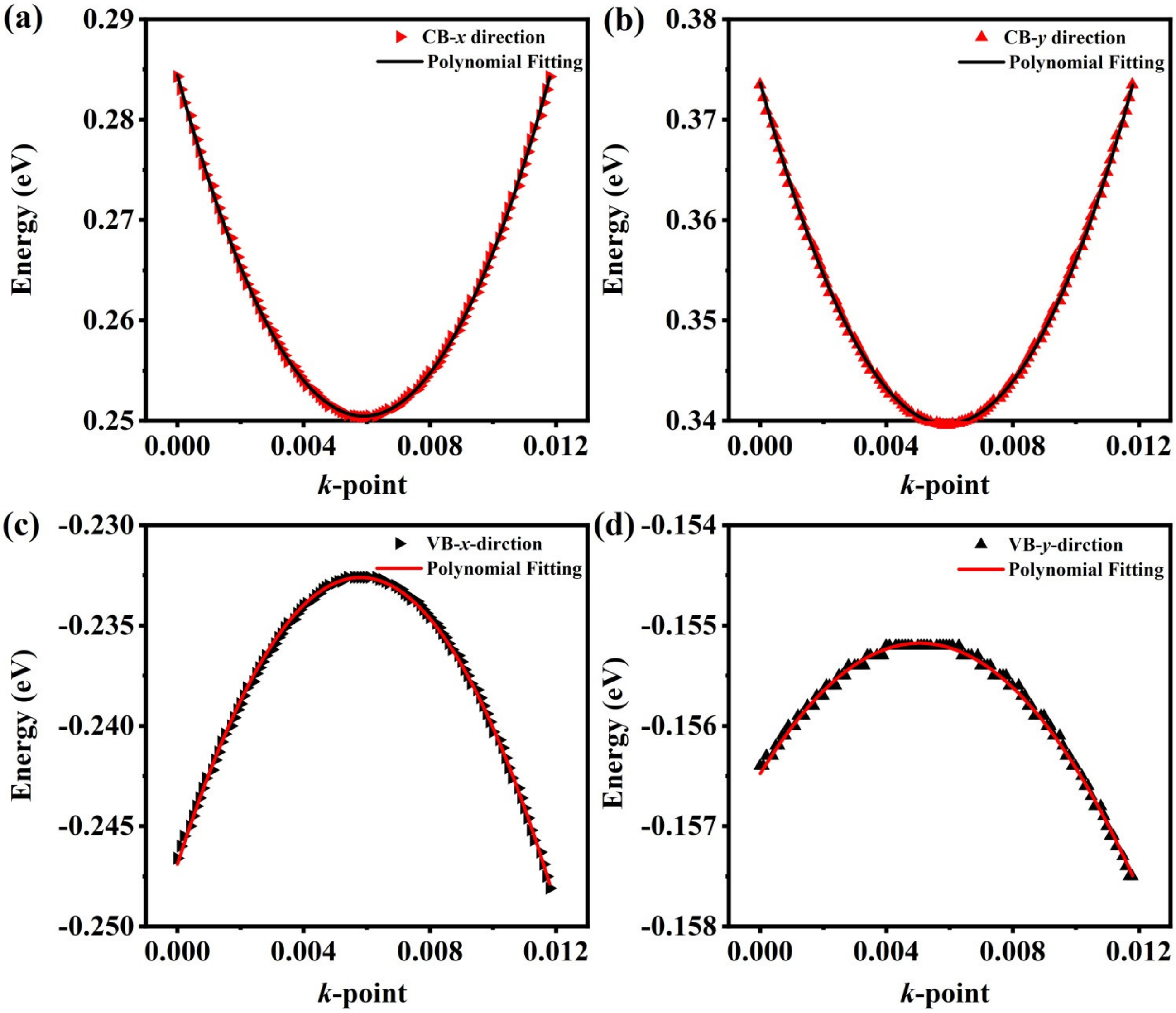

Figure S13. The quadratic fitting between the energy near the CBM (VBM) and k-point along the x and y directions under the biaxial strain of -1%.

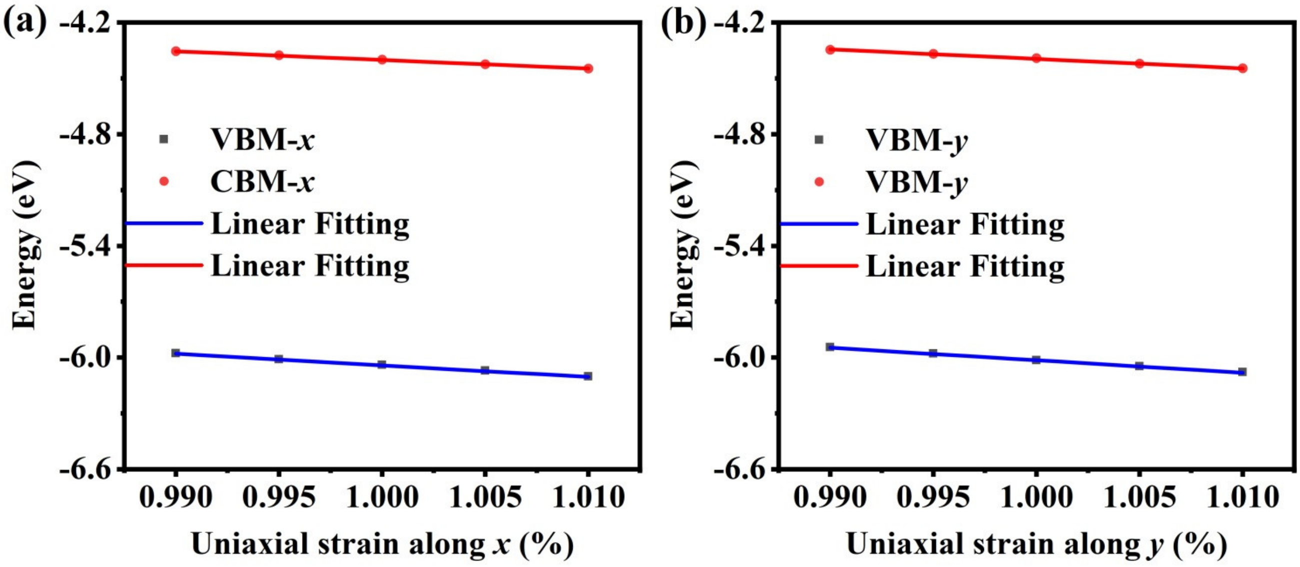

Figure S14. The linear fitting for the deformation potential constants along the x and y directions of single-layer $GaInSe_3$ under the biaxial strain of -1%.

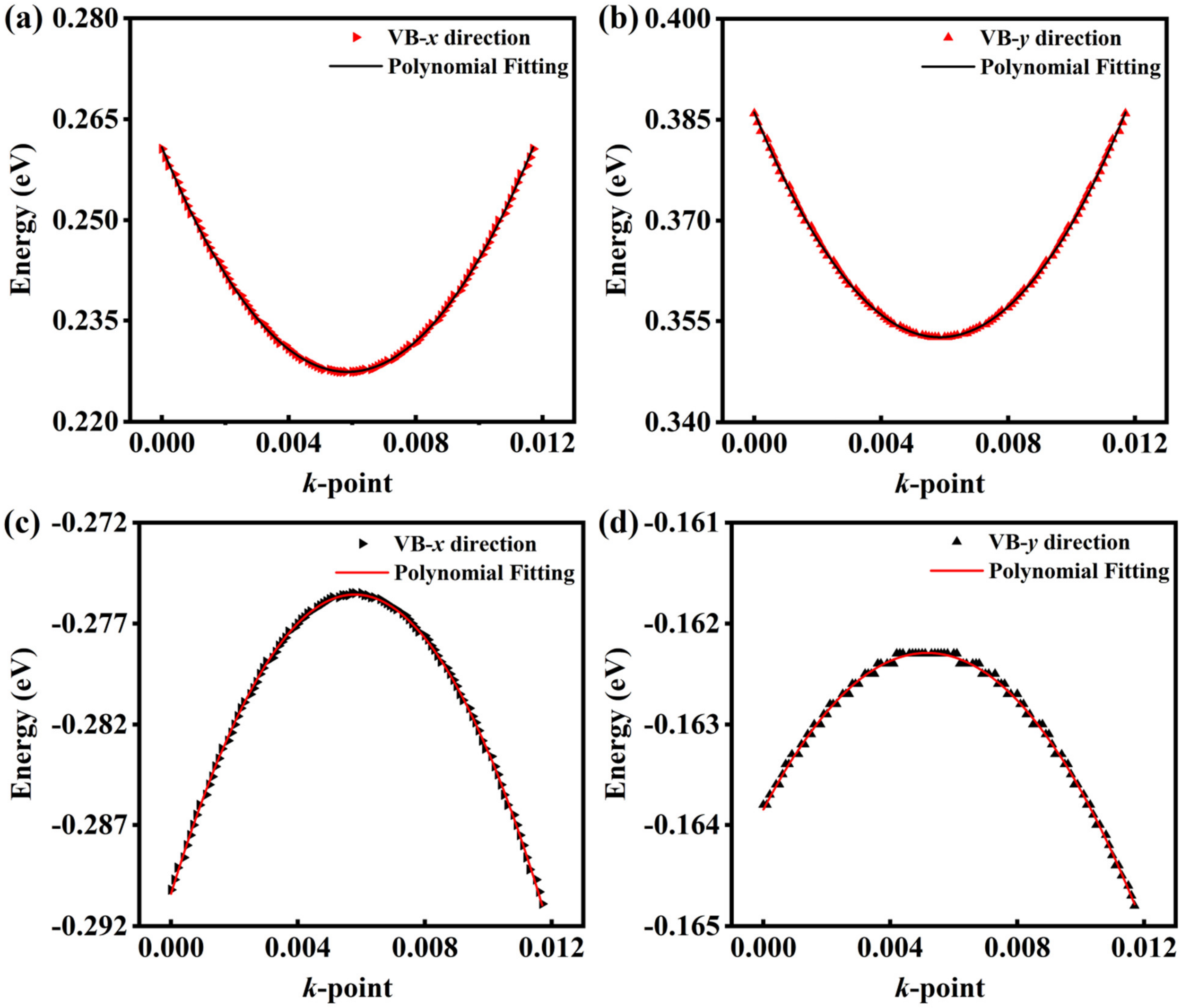

Figure S15. The quadratic fitting between the energy near the CBM (VBM) and k-point along the x and y directions under the biaxial strain of 0%.

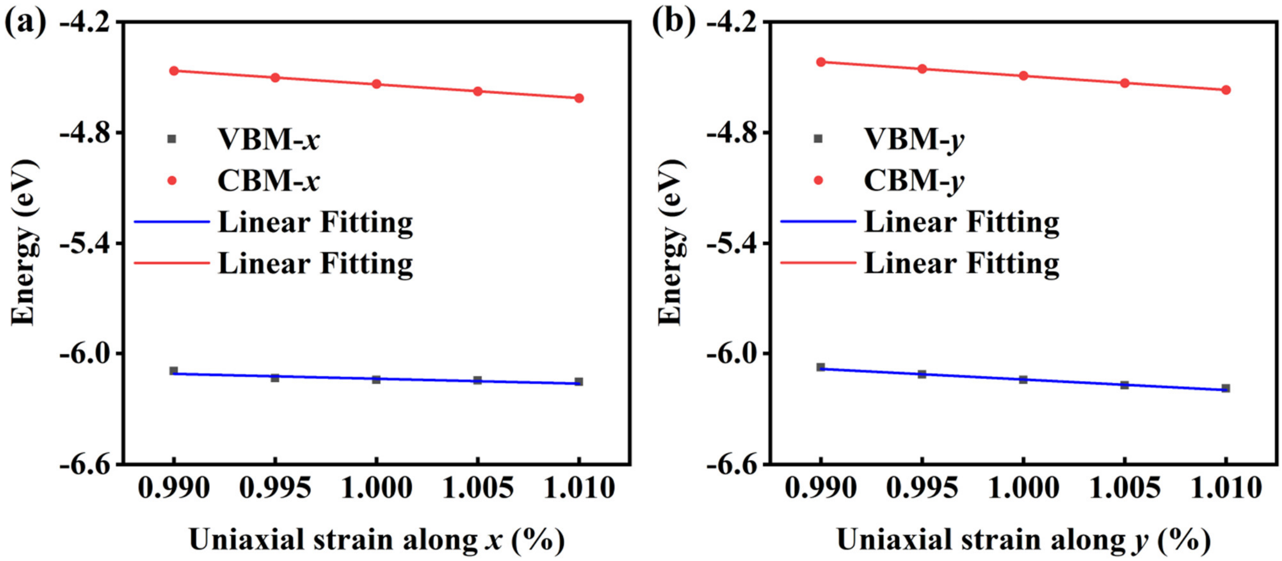

Figure S16. The linear fitting for the deformation potential constants along the x and y directions of single-layer $GaInSe_3$ under the biaxial strain of 0%.

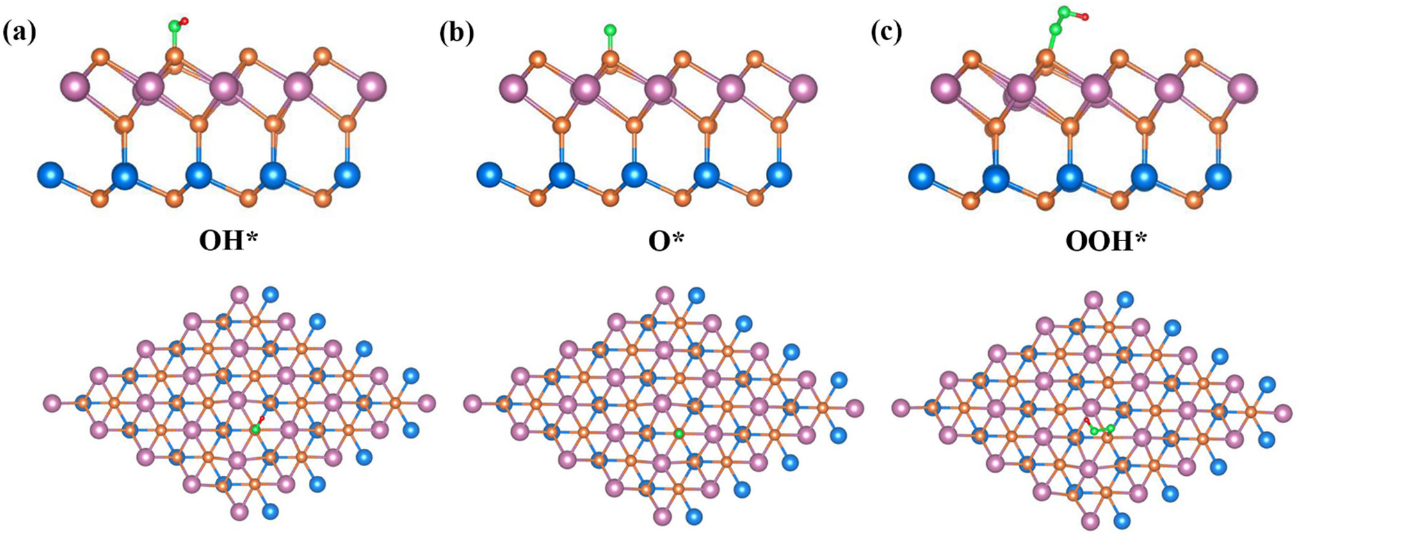

Figure S17. The side and top configurations of (a) OH*, (b) O*, and (c) OOH* intermediates of unstrained single-layer $GaInSe_3$ with a 4×4×1 supercell

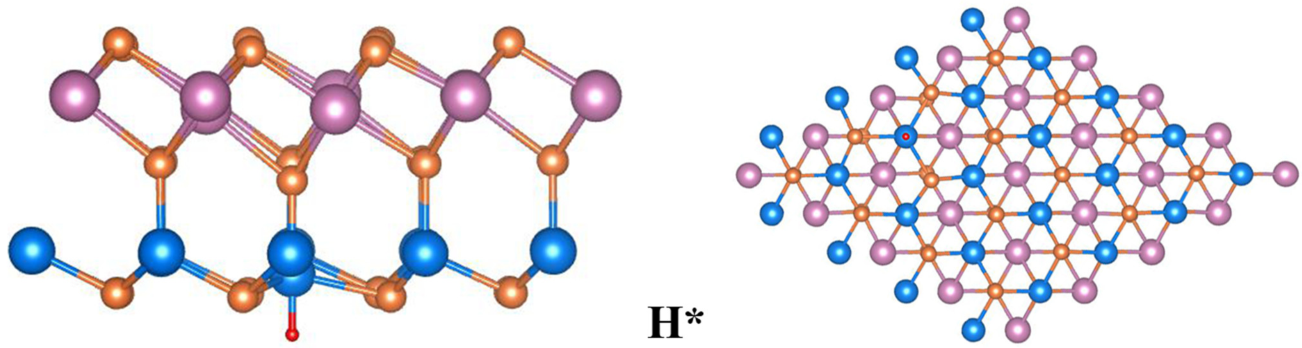

Figure S18. The side (Left) and top (Right) configuration of the H* intermediate of unstrained single-layer $GaInSe_3$ with a 4×4×1 supercell.

Table S1. The in-plane lattice constants a (Å), vacuum level difference between top and bottom surfaces $\Delta\Phi$ (eV), higher vacuum level Φ_h (eV), and lower vacuum level Φ_l (eV) of single-layer $GaInSe_3$.

Strain	a	$\Delta\Phi$	Φ_h	Φ_l
-2%	3.87	1.52	3.59	2.07
-1%	3.91	1.48	3.52	2.04
0%	3.95	1.44	3.46	2.02
+1%	3.99	1.42	3.39	1.97
+2%	4.03	1.36	3.33	1.97

Table S2. The calculated elastic constants C_{11} C_{12}, and C_{66} (N/m) of single-layer $GaInSe_3$ under different biaxial strains.

Strain	C_{11}=C_{22}	C_{12}=C_{21}	C_{66}
-2%	85.61	29.70	27.96
-1%	82.13	27.62	27.25
0%	78.36	25.80	26.28
+1%	74.17	24.27	24.95
+2%	69.56	22.87	23.35

Table S3. The DFT energy (E), zero-point energy correction (E_{ZPE}), entropy contribution (TS, T=300 K), and Gibbs Free Energy (G) of single-layer $GaInSe_3$.

Species	E (eV)	ZPE (eV)	TS (eV)	G (eV)
OH*	-318.615	2.412	9.323	-325.526
O*	-313.664	2.056	9.074	-320.682
OOH*	-322.795	2.547	9.237	-329.485
H_2O	-14.424	0.566	0.675	-14.533
H_2	-6.805	0.265	0.405	-6.945
*	-310.985	1.708	7.394	-316.671
H*	-310.934	2.253	9.021	-317.701
